# A GFP*-lacZ* Bicistronic Reporter System for Promoter Analysis in Environmental Gram-Negative Bacteria

**DOI:** 10.1371/journal.pone.0034675

**Published:** 2012-04-06

**Authors:** Rafael Silva-Rocha, Victor de Lorenzo

**Affiliations:** Systems Biology Program, Centro Nacional de Biotecnología, CSIC, Cantoblanco, Madrid, Spain; University of Minho, Portugal

## Abstract

Here, we describe a bicistronic reporter system for the analysis of promoter activity in a variety of Gram-negative bacteria at both the population and single-cell levels. This synthetic genetic tool utilizes an artificial operon comprising the *gfp* and *lacZ* genes that are assembled in a suicide vector, which is integrated at specific sites within the chromosome of the target bacterium, thereby creating a monocopy reporter system. This tool was instrumental for the complete *in vivo* characterization of two promoters, *Pb* and *Pc*, that drive the expression of the benzoate and catechol degradation pathways, respectively, of the soil bacterium *Pseudomonas putida* KT2440. The parameterization of these promoters in a population (using β-galactosidase assays) and in single cells (using flow cytometry) was necessary to examine the basic numerical features of these systems, such as the basal and maximal levels and the induction kinetics in response to an inducer (benzoate). Remarkably, GFP afforded a view of the process at a much higher resolution compared with standard *lacZ* tests; changes in fluorescence faithfully reflected variations in the transcriptional regimes of individual bacteria. The broad host range of the vector/reporter platform is an asset for the characterization of promoters in different bacteria, thereby expanding the diversity of genomic chasses amenable to Synthetic Biology methods.

## Introduction

The process of gene regulation in a living organism is vitally important for its adaptation to changing conditions in the environment. In fact, the analysis of the complete genomes currently available reveals that a large amount of the genome encodes sequences related to gene regulation and transcription [1], [2], [3], [4], [5]. In the case of prokaryotes, the comparison between organisms with different lifestyles shows that the generalists (such as free living environmental bacteria) usually have a higher proportion of their genome content dedicated to gene regulation than the specialists (e.g., endosymbionts, [6]). Moreover, the coordination of gene expression involves several steps that are controlled by transcriptional factors (TFs, [7], [8], [9]). In the last few years, there has been tremendous progress in the study of regulatory networks, and evidence has shown that, among other things, gene expression is also largely susceptible to stochastic variations from cell to cell [10], [11], [12], primarily because there are typically few units of the reactant molecules (TF, promoters, etc.) found in the cell cytoplasm [12], [13], [14]. The biochemical processes underlying gene regulation are driven by the collision of the reactants; therefore, the low amount of the reactants makes the system prone to a higher level of noise in the final output. In addition, reactions endowed with low kinetic constants contribute to the increased noise of the cell [11].

While living organisms can indeed use noise to control crucial differentiation programs, such as competence in *Bacillus subtilis* [15], [16], it is usually deleterious for the proper functioning of intracellular circuits. In fact, the improper function of some synthetic circuits in bacteria is a consequence of high levels of noise during the different processes of gene regulation [17], [18], [19], [20]. For the integration of stochasticity in decision-making switches, the resulting network architectures are usually associated with the generation of multi-stability, where it is possible for the cell to obtain multiple stable states [16], [21], [22]. In a multi-stable system, the presence of noise is crucial as small fluctuations during gene regulation determine the fate of the network and thus its final steady state [16], [21], [23].

An analysis of the stochastic process in cellular systems is fundamental not only for the understanding of the underlying process in a given regulatory network, but also for the proper characterization of molecular components that regulate synthetic circuits [24], [25], [26]. Thus, single-cell methodologies based on fluorescent proteins, such as GFP, are crucial for the analysis of noise. While classical approaches based on the enzymatic quantification of a reporter gene (e.g., *lacZ*) only provide information concerning the gene expression process within a population, fluorescent reporter assays offer high resolution information at the single cell level [11]. Ideally, a single reporter system should be instrumental to examine both of these aspects, but indicator products (e.g., fluorescent proteins) that are best suited for use in single cells perform poorly in cultures because of quenching and other optical interferences. Similarly, the best population-level reporters (e.g., *lacZ* and *luxAB*) give diffuse signals that prevent the examination of particular bacteria. One possible solution is the combination of two reporter products in a single transcriptional unit [27], [28] or hybrid functional polypeptide [29]. Unfortunately, these constructs are only amenable for model bacteria, such as *E. coli*, and the toolbox for less standardized microorganisms, such as environmental Gram-negative bacteria, is much more scarce [30], [31]. For instance, the soil bacterium *Pseudomonas putida* KT2440 is endowed with the remarkable capability to degrade aromatic compounds [31], [32]. As this organism is naturally adapted to deal with toxic pollutants, it is a more versatile bacterium for *in situ* applications, such as biosensing xenobiotics [33], [34] and the mineralization of chemical contaminants. Yet, the number of tools available for studying gene expression in this bacterium –as well as in other Gram-negative microorganisms, is scarce as compared to *E. coli.*

In this paper, a dual promoter probe system based on the expression of GFP and *lacZ*, the two most widely used reporters in bacteria [35], is used for the examination of environmental Gram-negative bacteria. The system is designed to allow the insertion of an artificial operon (GFP-*lacZ*) into a specific position in the bacterial chromosome, creating a stable monocopy reporter system. The resulting strains can be then assayed for both GFP and *lacZ* expression, allowing the characterization of the target promoters in monocopy gene dose and stoichiometry. This system has been instrumental for studying the regulation of the benzoate and catechol degradation pathways in *P. putida*, which show monostable behavior during the entire catabolic process. We anticipate that the applications of this system will be expanded from the fundamental research of regulatory networks to more applied endeavors in Synthetic Biology.

## Results and Discussion

### Construction of a Dual GFP-*lacZ* Reporter System

**Figure 1:**
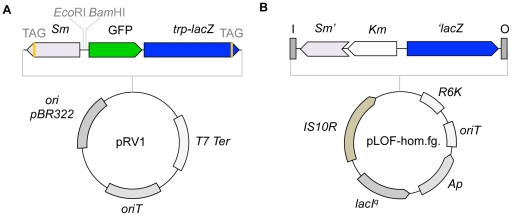
Bicistronic reporter system based on GFP and *lacZ*. **A** The reporter vector pRV1 containing truncated versions of the *lacZ* gene and Sm/Sp resistance marker could only be maintained in *E. coli* strains with *supF* tRNA. GFP is located upstream of the *lacZ* gene and is preceded by the *Eco*RI/*Bam*HI cloning site for the target promoters. The *oriT* sequence allows the mobilization of pRV1 to new hosts by conjugation. **B** The mini-Tn10-based vector pLOF-hom.fg. was used for target strain modification. This vector contains the homologous fragment, which was introduced into the strains of interest to create single copy insertions in the chromosome.

Initially, we created a bicistronic system for promoter probing based on the expression of GFP and *lacZ* genes using as a reference a system for the single copy integration of a *lacZ* reporter that was previously described by Kessler and co-workers [36]. Briefly, a stable version of GFP [37] and a modified version of the *lacZ* gene, which had a premature amber stop codon TAG at the 3' region, was inserted into the suicide vector pRV1 harboring a promoterless synthetic operon (Fig. 1A; [36]). A streptomycin/spectinomycin (Sm/Sp) resistance marker, which was located in a divergent orientation, also contained a premature stop codon (Fig. 1A; [36]). The presence of the amber codons made the two resulting proteins non-functional unless the host strain harbored a *supF* tRNA. In addition, because of the narrow host range of pBR322 *ori*, the pRV1 vector is unable to replicate in organisms unrelated to *E. coli* [36]. Moreover, the RP4 *oriT* transfer origin allowed for plasmid mobilization through conjugation to target bacterium, such as *P. putida*. The target promoter is cloned into the pRV1 vector using *Eco*RI and *Bam*HI restriction enzyme sites located upstream of the GFP gene (Fig. 1A). To integrate the GFP-*lacZ* reporter cassette, the target strain should contain a homologous fragment in the chromosome to mediate double homologous recombination (see below, [36]). The mini-Tn*10*-based transposon pLOF-hom.fg. was used to modify the transferred strain. In this transposon, a homologous fragment containing the *lacZ* gene, which lacked the ATG start codon, and a Sm/Sp marker disrupted by a kanamycin (Km) gene were placed in the transposable element (Fig. 1B). As explained in the next section, recombination between the versions of the Sm marker and the *lacZ* gene allowed the integration of the reporter system through the reconstitution of fully functional genes in the chromosome.

### Generation of a Non-fluorescent *P. Putida* Strain

**Figure 2:**
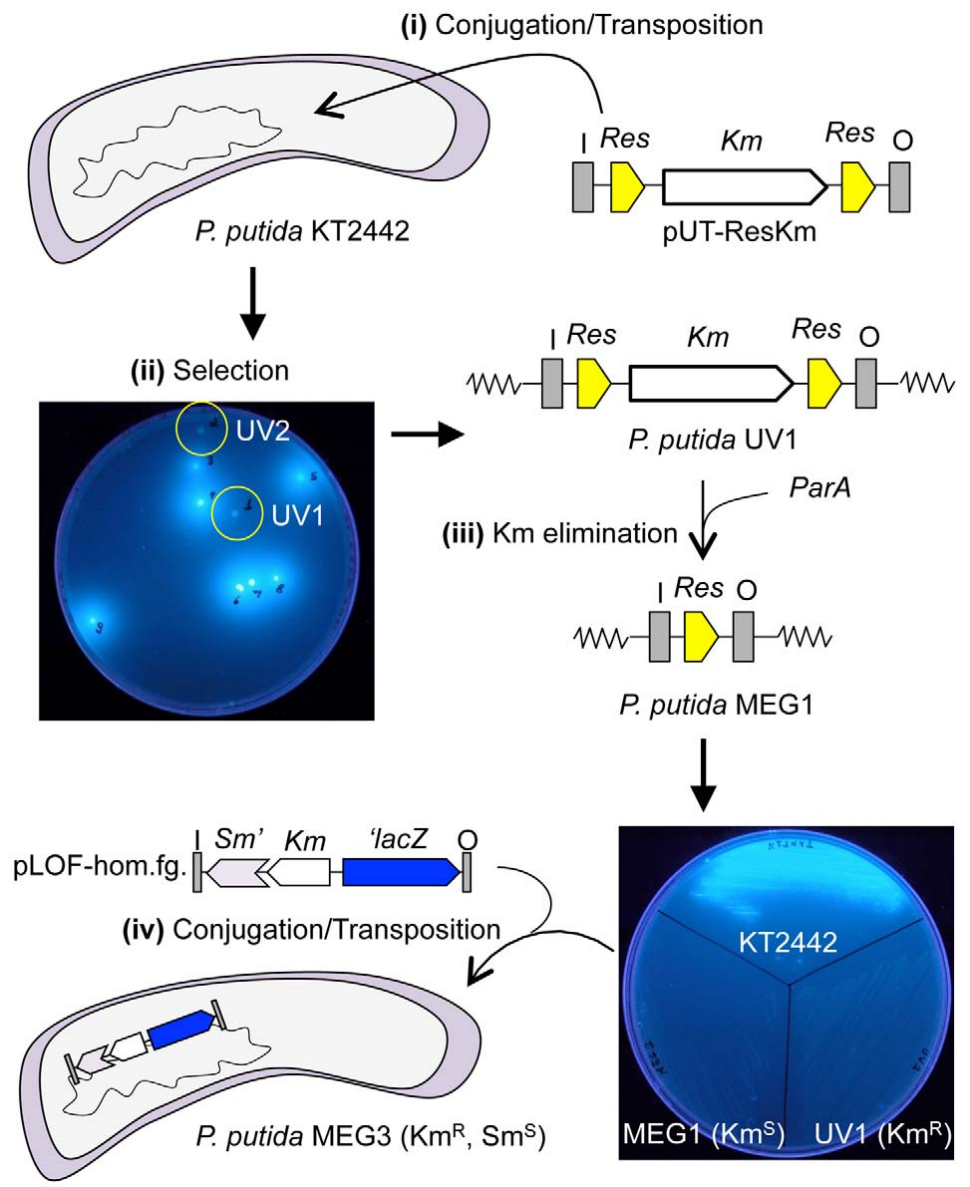
Isolation of a non-fluorescent *P. putida* strain. **(i)** *P. putida* KT2442, a rifampicin resistance variant of *P. putida* KT2440, was mutagenized with mini-Tn5 transposon pUT-ResKm. **(ii)** Single colonies were selected in minimal media and screened for the lack of fluorescence. Two colonies were selected and named *P. putida* UV1 and UV2. **(iii)** The Km resistance marker was removed from the strains by expressing ParA resolvase, which recognizes the two *res* sites flanking the Km marker. After marker elimination, the strains retained the non-fluorescence phenotype. **(iv)** The homologous fragment placed in the mini-Tn10 transposon was mobilized to the marker-less *P. putida* MEG1 strain (a deviant of *P. putida* UV1), generating the *P. putida* MEG3 strain. This strain was used as a host for the bicistronic reporter system with the target promoters.

The soil bacterium *P. putida* produces a fluorescent siderophore pyoverdine for the capitation of iron ions from the environment [38], [39]. To assay for GFP expression in *P. putida*, we generated a bacterial strain that was unable to produce pyoverdine, as this siderophore may mask the fluorescence from GFP. The steps for mutagenization of *P. putida* are summarized in Figure 2. First, a random mutant library was created using a mini-Tn*5*-based vector containing a removable Km marker (Fig. 2, step I). The pyoverdine production was assayed in minimal media plates under ultraviolet (UV) light, and the two colonies that were unable to fluoresce were selected and named *P. putida* UV1 and UV2 (Fig. 2, step II). Subsequently, the Km resistance markers of the two strains were removed by expressing the resolvase coding gene *parA*, generating strains *P. putida* MEG1 and MEG2 (derived from *P. putida* UV1 and UV2, respectively). As shown in Figure 2, upon removal of the Km marker, the strains did not recover the fluorescent phenotype (step III). Once we generated bacterial strains suitable to analyze GFP, we introduced the homologous fragment into the chromosome of *P. putida* by targeting *P. putida* MEG1 with the pLOF-hom.fg. transposon as indicated in the Materials and Methods section. The resulting strain, harboring the homologous fragment stably integrated in the chromosome, was named *P. putida* MEG3 and used in further analyses.

### Assembly of Monocopy Promoter Fusions to the GFP-*lacZ* System

**Figure 3:**
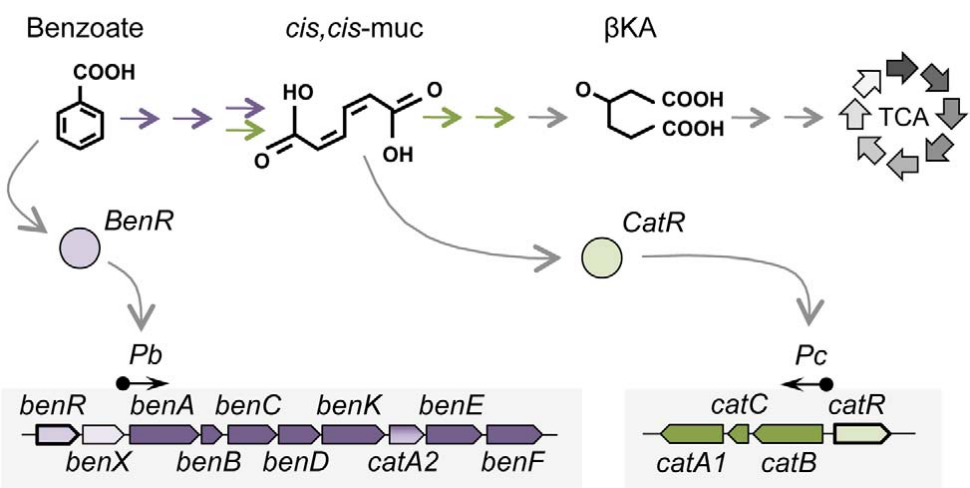
Catabolic pathway for benzoate degradation in *P. putida*. Benzoate activates the BenR regulator, which controls the expression of the *ben* operon. The enzymes encoded by the *ben* operon metabolize the conversion of benzoate to *cis,cis*-muconate (*cis,cis*-muc), which is the signal for CatR activation. CatR controls the expression of the *cat* operon for the metabolization of *cis,cis*-muconate, which is further converted to β-ketoadipate. Finally, β-ketoadipate is converted to TCA intermediates through the action of the *pca* pathway (gray arrows).

As previously discussed, *P. putida* is endowed with the remarkable capability to degrade a number of aromatic compounds [32]. In this organism, benzoate is degraded through the β-ketoadipate pathway, which is highly distributed among the *Pseudomonas* genera and related organisms [40]. In this pathway, benzoate is oxidized to catechol, which is then subjected to an intradiol cleavage to generate *cis,cis*-muconate (*cis,cis*-muc, Fig. 3). Subsequently, *cis,cis*-muconate is degraded to form β-ketoadipate, which is further degraded to tricarboxylic acid cycle (TCA) intermediates. The first steps of benzoate metabolization are performed for the enzymes encoded in the *ben* operon [40]. This operon is expressed from the *Pb* promoter, which is activated by the AraC-type regulator BenR bound to benzoate (Fig. 3; [41]). The metabolization of *cis,cis*-muconate depends upon the action of the *cat* genes. These genes are activated by the LysR-type regulator CatR, a TF that responds to the presence of *cis,cis*-muconate [42], [43]. Finally, the final steps of metabolization are performed by the *pca* genes, which are controlled by the β-ketoadipate-induced IclR-type regulator PcaR [40], [44].

**Figure 4:**
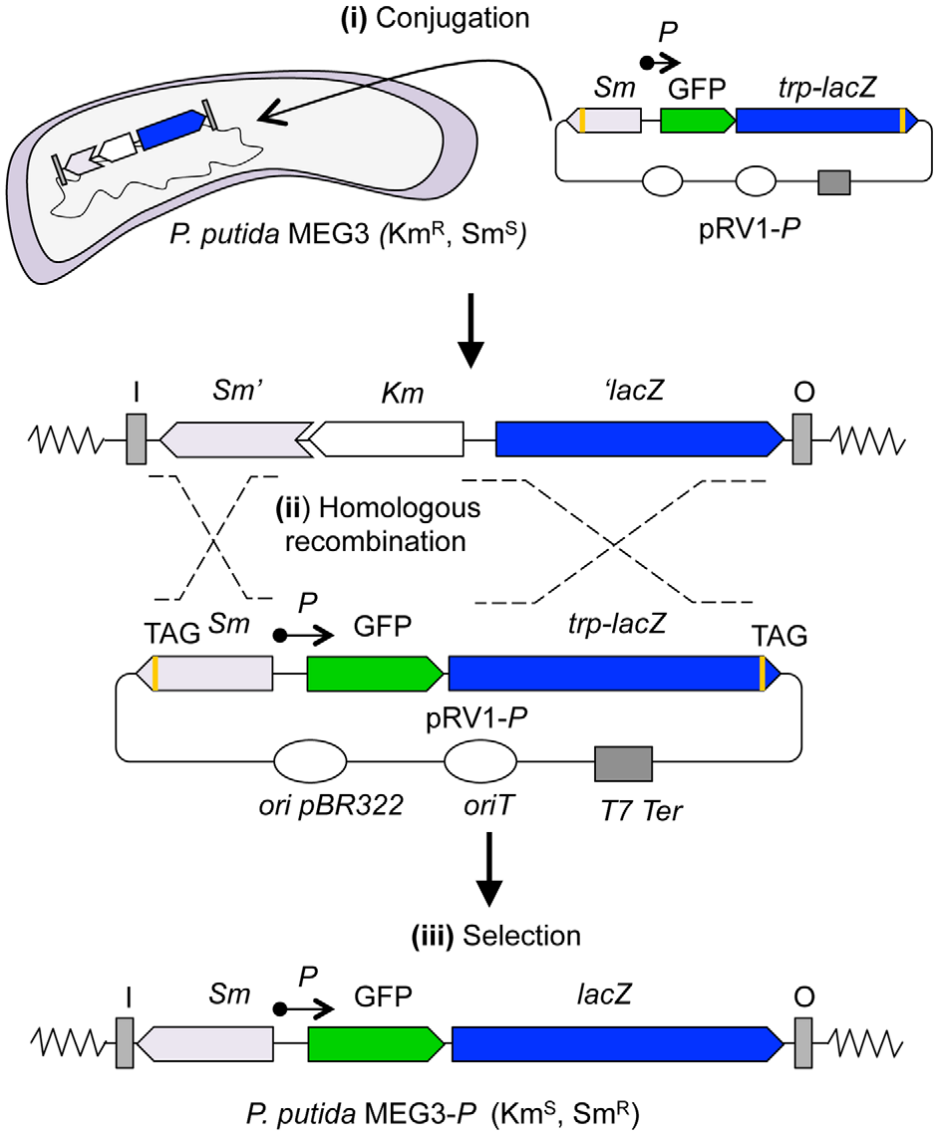
Design of bicistronic GFP-*lacZ* reporter strains. **(i)** pRV1 containing the cloned promoters (labeled as *P*) was introduced into the * P. putida* MEG3 strain by conjugation. Colonies were selected in minimal media using the Sm antibiotic. **(ii)** The homology regions of the suicide vector pRV1 were recombined with the two segments placed in the chromosome, generating a functioning Sm/Sp resistance marker and *lacZ* gene. **(iii)** Finally, the correct insertion of the segments generated a strain with a stable reporter system that was sensitive to the Km antibiotic.

To validate the performance of the bicistronic reporter system described here, we analyzed the dynamic properties for the initial steps that control the expression of the β-ketoadipate pathway in *P. putida*. First, the promoter regions of the *ben* and *cat* operons were cloned into the pRV1 reporter vector. The resulting constructs where introduced into *P. putida* MEG3 by tri-parental mating as previously described (Fig. 4; [45]). After plasmid transference to *P. putida*, the cells were selected in minimal media using Sm/Sp as the resistance marker. Following two homologous recombination events, a fully functional Sm/Sp marker and *lacZ* gene were generated in the chromosome of the recipient bacterium, and the Km resistance phenotype of the strain was lost (Fig. 4). The resulting strains containing the correct insertion of the *Pb*- and *Pc*-based systems were named *P. putida* MEG3-*Pb* and MEG-*Pc*, respectively, and used in further assays.

### Population and Single-cell Analysis of Promoter Activity in *P. Putida*

**Figure 5:**
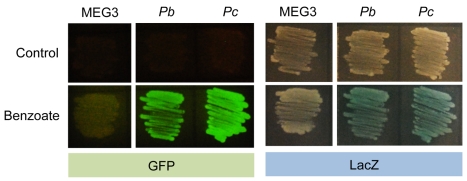
Dual reporter expression in *P. putida* MEG3 strains. GFP expression is shown on the left, while *lacZ* expression is shown on the right. The strain with no promoter cloned (labeled as MEG3) was used as a control. The strains having *Pb* and *Pc* fused to the dual reporter system presented GFP and LacZ signals when cultured in the presence of 1 mM benzoate.

**Figure 6:**
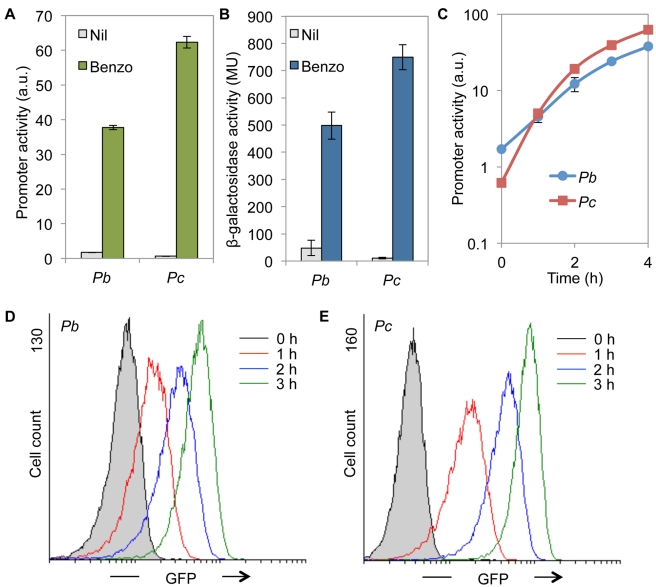
Assay of promoter activities in response to benzoate. Overnight cultures were diluted in fresh minimal media supplemented with succinate as the sole carbon source. At the mid-exponential phase, the cells were exposed to 1 mM benzoate, and the GFP and the *lacZ* expression was assayed. **A** The GFP expression was measured in *P. putida* MEG3-*Pb* and MEG3-*Pc* after 4 hours of induction using flow cytometry. **B** β-galactosidase activity of *P. putida* MEG3-*Pb* and MEG3-*Pc* after 4 hours of induction. **C** Time course quantification of GFP expression in *P. putida* MEG3-*Pb* and MEG3-*Pc* in response to 1 mM benzoate. The vertical bars represent the standard deviation of the experiments performed in duplicate. **D** The quantification of GFP levels in the *P. putida* MEG3-*Pb* population in response to 1 mM benzoate. The distribution of cell fluorescence is shown along the induction curve. At each time point, 15,000 events were analyzed. **E** The quantification of GFP levels in the *P. putida* MEG3-*Pc* population in response to 1 mM benzoate. The experiments were performed as in **D**.

After constructing *P. putida* reporter strains containing monocopy bicistronic cassettes, we investigated the expression profiles of *Pb* and *Pc* in response to benzoate. As shown in Figure 5, *P. putida* MEG3-*Pb* and MEG-*Pc* simultaneously expressed GFP and LacZ proteins in the presence of benzoate on the agar plates. To quantify the promoter activity in response to benzoate present in the liquid media, an overnight culture of cells was diluted in minimal media containing succinate as the sole carbon source and incubated for a few hours. At the mid-exponential phase, 1 mM benzoate was added to the growth media, and the cells where incubated for an additional 4 hours. Subsequently, samples were taken and analyzed using flow cytometry to quantify GFP expression, and a β-galactosidase assay was used to quantify the expression of *lacZ*. As shown in Figure 6A, *Pb* and *Pc* showed a higher level of induction in response to benzoate as assayed using GFP. In general, *Pb* presented a higher basal level and a lower maximal activity compared with *Pc* (Fig. 6A). Interestingly, the analysis of the promoter activities using the β-galactosidase assay showed the same overall results, indicating that the synthetic construction worked faithfully as a bicistronic unit (Fig. 6B). The primary difference between the two reporters was observed in the fold-change detected in the activity of the two promoters depending on the reporter examined. In the case of GFP, a 22-fold induction of *Pb* was observed, whereas *Pc* presented a fold change of 100. However, when *lacZ* was used as the reporter, we observed a fold induction of 10 and 67 for *Pb* and *Pc*, respectively. These results indicated that, under the conditions specified, GFP provides a higher resolution of the changes in transcription rates in response to the inducer. This circumstance seems to improve the signal-to-noise ratio and thus provides a systematically higher induction as compared with that of *lacZ* in the same cells, where both reporters are expressed simultaneously.

We next analyzed the behavior of the two promoters in a single cell using GFP. The promoter activities were monitored along the induction curve using flow cytometry as described in the Materials and Methods section. As shown in Figure 6C, at the population level, the *Pc* promoter showed a more noticeable induction curve than *Pb*, as the former started at a lower basal value and reached a higher maximal activity. When we analyzed the distribution of fluorescence from cell to cell within the population, we found that both promoters presented similar dynamic behaviors for GFP expression. Only single populations were observed at each time point, and a continuous transition in the fluorescence levels was observed (Fig. 6D-E). These results suggested that a mono-stable regulatory device controls the β-ketoadipate pathway for benzoate metabolism in *P. putida* which prevents the appearance of inactive subpopulations during biodegradation.

### Conclusion

Here, we describe the implementation and validation of a dual bicistronic reporter system based on the GFP and *lacZ* genes. This novel tool permits the characterization of the regulatory networks in many environmental bacteria for which only few genetic tools are available. By integrating the system in monocopy in the chromosome of the target bacterium, we avoided the deleterious effects associated with the stochastic variation of copy number observed in plasmid-based systems [17], [18], [19]. In fact, the noise generated in plasmid-based systems might interfere with the accurate analysis of the target network [46]. While the Tn*7* transposon-based system also provide an alternative to the insertion of synthetic constructs in single copy, this method only utilizes native sites existing in the bacterium [47]. However, the novel system introduced here exploits different genomic locations, as the integration site can be randomly generated using a mini-Tn*10* delivery transposon [48]. In addition, the combinatory use of this system with existing Tn*7*-based tools would permit the construction of stable strains with isogenic modifications, as different inserts would be placed at specified positions. We used this system to investigate the expression of the pathway for benzoate metabolism in the soil bacterium *P. putida*. Usually, the genetic characterization of the regulatory networks in this and other atypical organisms is performed in the model organism *E. coli* [49], [50], but this approach might miss important host-specific features. For example, using the new tool in the native host, we were able to monitor *cat* pathway expression in response to benzoate. Under the experimental conditions used in this study, benzoate was converted to *cis,cis*-muconate to trigger *Pc* activity (Fig. 3). Moreover, the novel system presented here can be easily applied to other organisms and would provide valuable information on the dynamics of regulatory networks and the implementation of new circuits in bacteria.

## Materials and Methods

### Bacterial Strains

*E. coli* CC118 [45] or its variant CC118*supF* [36] were used as the hosts for the plasmid constructs. *E. coli* HB101 (pRK600) was used as a helper strain for tri-parental mating as previously described [45]. The induction experiments were performed in M9 minimal medium [51] with 2 mM MgSO_4_ and 25 mM of succinate as the sole carbon source. This minimal medium was additionally supplemented with Sm (50 µg ml^–1^) and Km (50 µg ml^–1^) to ensure plasmid retention. The benzoate was purchased from Sigma-Aldrich.

### Plasmid Construction

For cloning purposes, DNA fragments were amplified using the polymerase chain reaction (PCR) containing 50–100 ng of the template, 50 pmol of each primer and 2.5 U of *Pfu* DNA polymerase (Stratagene) in a 100 µl reaction volume. The mixtures were subjected to 30 cycles of 1 min at 95°C, 1 min at 55°C and 2 min at 72°C. The primers used were purchased from Sigma. The plasmid DNA purification, restriction enzyme digestion and general cloning procedures were conducted using standard protocols [51]. For the analysis of the stochastic effects of the TOL promoters under inducing conditions, we developed a system for the insertion of transcriptional GFP fusions in the chromosome of *P. putida*. This system is a modified version of a *lacZ*-based system described by Klesser *et al.*, which comprises a suicide vector pBK16 (Fig. 1A) and a homology fragment placed in a mini-Tn*10* transposon (Fig. 1B, [36]). To generate a variant of pBK16 having a fluorescent reporter, the *gfp tir* gene from the pGREENtir [37] plasmid was PCR amplified using the primers 5GFP (5'-GAA TTC ATC GGA TCC TGA TTA ACT TTA TAA GGA GG-3') and 3GFP (5'-ATA GAT CTT TAT TAT TTG TAT AGT TCA TCC ATG CC-3'). The resulting PCR fragment was digested with the restriction enzymes *Eco*RI and *Bgl*II and cloned into a pBK16 vector that was previously digested with *Eco*RI and *Bam*HI. The resulting vector pRV1 performed as a bicistronic *gfp*/*lacZ* reporter system (Fig. 1A). The promoters of the *ben* (*Pb*) and *cat* (*Pc*) pathways where PCR amplified using the primer pairs PBf (5'-TGG ATG AAT TCG ACA GTA CCC TCC-3')/PBr (5'-GCG CGG ATC CGG CCA GGG TCT CCC TTG-3') and PCf (5'-GAG AGA ATT CAG GCC CAG TTC CAG CTC G-3')/PCr (5'-GCG CGG ATC CTG TTG CCA GGT CCC GTC AG-3'), respectively, and cloned as *Eco*RI/*Bam*HI fragments into pre-digested pRV1 vectors. The resulting reporter vectors were introduced into *P. putida* MEG3 as described below.

### Construction of the Non-fluorescent *P. Putida* Strains

To perform experiments using GFP in *P. putida*, we generated variants of this organism that were unable to auto-fluorescence. Briefly, overnight cultures of *P. putida* KT2442 were mutagenized with the pUT-ResKm transposon (a modified version of pUT-Km [52] with *res* sites flanking the Km marker) by tri-parental mating [45]. The *P. putida* transposition library was plated on M9 agar plates supplemented with 0.2% citrate and Km. After overnight incubation, the colonies were screened under ultraviolet (UV) light at 254 nm for the identification of strains with no auto-fluorescence (Fig. 2). Two strains that did not fluoresce under UV light were selected and named *P. putida* UV1 and UV2. Subsequently, the plasmid pJMSB8 [53] was transferred to *P. putida UV1* and *P. putida* UV2 by tri-parental mating. This plasmid expressed the gene for the ParA enzyme, which catalyzes the recombination between adjacent Res sequences [53]. The pUT-ResKm transposon used to create strains UV1 and UV2 contained a Km gene flanked by two *res* sequences, thus the expression of ParA in this system expression eliminated the expression of the resistance marker. Upon insertion of the ParA coding plasmid, single colonies of strains UV1 and UV2 were analyzed for the loss of resistance to Km. Two strains demonstrating successful antibiotic removal were confirmed and named *P. putida* MEG1 and MEG2 (derived from UV1 and UV2, respectively) and used for further analysis (Fig. 2).

### Construction of the *P. Putida* MEG3 Reporter Strains

For the analysis of the TOL promoter activities at the single cell level, we used a modified version of the pBK16 homologous recombination system (see above, [36]). To generate a *P. putida* variant that was able to accommodate the GFP reporter system in the chromosome, we first mutagenized *P. putida* MEG1 using the pLOF-hom.fg. mini-Tn*10* transposon [36]. This transposon contains a *lacZ* gene lacking the ATG start codon cloned in the opposite direction of an Sm/Sp resistance marker truncated by the insertion of a Km gene (Fig. 1B). The resultant transposon library was plated on M9 minimal media supplemented with 0.2% citrate and Km. A single colony that was able to grow in Km and confirmed as the resultant of two transposition events was named MEG3 and used in further experiments. For the generation of *P. putida* strains containing transcriptional fusions to GFP in the chromosome, pRV1-variants with different promoters were transferred to *P. putida* MEG3 (Fig. 4). After tri-parental mating, the strains were plated on M9 minimal media supplemented with 0.2% citrate and Sm to select for homologous recombination events between the Sm/Sp gene from the pRV1 vector and the homologous fragments placed in the chromosome of MEG3 strain (Fig. 4). As a control, a *P. putida* strain lacking the homology fragment was used as the recipient for conjugation. No Sm-resistant colonies were observed, ruling out the possibility for plasmid integration on alternative sites. Finally, single colonies were assessed for the second recombination event (*i.e.*, between the two variants of the *lacZ* gene) by analyzing their sensitivity to Km. Strains containing the correct insertion of the reporter system in the chromosome were named *P. putida* MEG3-*P* (where *P* stands for the identity of the cloned promoter) and used for the single-cell analysis.

### GFP Analysis at the Single-cell Level

For the quantification of GFP expression at the single-cell level, *P. putida* MEG3 strains containing different promoter fusions to GFP were inoculated into M9 media supplemented with 25 mM succinate. After overnight growth, the cultures were washed twice, diluted 1∶20 in fresh M9 media containing 25 mM succinate and incubated for an additional 4 hours. After this pre-incubation, the cultures were distributed into new flasks containing different concentrations of benzoate and incubated with air shaking. Each hour after induction, 500 µL samples were spun down, the cells resuspended in 500 µL of PBS and stored on ice until analysis. The GFP distribution in the cell population was analyzed by flow cytometry using a GALLIOS cytometer (Perkin Elmer). For each sample, 15,000 events were analyzed. The data processing was performed using Cyflogic software (http://www.cyflogic.com/). Calculations of the mean fluorescence from the different replicas and standard deviations were calculated with the statistical package of Microsoft Excel (2010).

### β-galactosidase Assay

To perform the LacZ activity assay, single colonies of reporter strains were grown overnight in M9 media supplemented with 25 mM succinate at 30°C. The overnight cultures were subsequently diluted 1∶20 in fresh M9 media containing 25 mM succinate and cultured for an additional 4 hours. Subsequently, benzoate was added to the media, and the cells were incubated for several hours. β-galactosidase activities were assayed in permeabilized whole cells according to Miller’s method with minor modifications [54].

## References

[1] Itzkovitz S, Tlusty T, Alon U (2006). Coding limits on the number of transcription factors.. BMC Genomics.

[2] Teichmann SA, Babu MM (2004). Gene regulatory network growth by duplication.. Nat Genet.

[3] Konstantinidis KT, Tiedje JM (2004). Trends between gene content and genome size in prokaryotic species with larger genomes.. Proc Natl Acad Sci USA.

[4] Martinez-Antonio A, Collado-Vides J (2003). Identifying global regulators in transcriptional regulatory networks in bacteria.. Curr Opin Microbiol.

[5] Thieffry D, Huerta AM, Perez-Rueda E, Collado-Vides J (1998). From specific gene regulation to genomic networks: a global analysis of transcriptional regulation in *Escherichia coli.*. Bioessays.

[6] Cases I, de Lorenzo V, Ouzounis CA (2003). Transcription regulation and environmental adaptation in bacteria.. Trends Microbiol.

[7] Silva-Rocha R, de Lorenzo V (2010). Noise and robustness in prokaryotic regulatory networks.. Ann Rev Microbiol.

[8] Balleza E, Lopez-Bojorquez LN, Martinez-Antonio A, Resendis-Antonio O, Lozada-Chavez I (2009). Regulation by transcription factors in bacteria: beyond description.. FEMS Microbiol Rev.

[9] McAdams HH, Srinivasan B, Arkin AP (2004). The evolution of genetic regulatory systems in bacteria.. Nat Rev Genet.

[10] Pedraza JM, van Oudenaarden A (2005). Noise propagation in gene networks.. Science.

[11] Elowitz MB, Levine AJ, Siggia ED, Swain PS (2002). Stochastic gene expression in a single cell.. Science.

[12] McAdams HH, Arkin A (1997). Stochastic mechanisms in gene expression.. Proc Natl Acad Sci USA.

[13] Raj A, van Oudenaarden A (2008). Nature, nurture, or chance: stochastic gene expression and its consequences.. Cell.

[14] McAdams HH, Arkin A (1999). It’s a noisy business! Genetic regulation at the nanomolar scale.. Trends Genet.

[15] Cagatay T, Turcotte M, Elowitz MB, Garcia-Ojalvo J, Suel GM (2009). Architecture-dependent noise discriminates functionally analogous differentiation circuits.. Cell.

[16] Maamar H, Dubnau D (2005). Bistability in the *Bacillus subtilis* K-state (competence) system requires a positive feedback loop.. Mol Microbiol.

[17] Elowitz MB, Leibler S (2000). A synthetic oscillatory network of transcriptional regulators.. Nature.

[18] Becskei A, Serrano L (2000). Engineering stability in gene networks by autoregulation.. Nature.

[19] Gardner TS, Cantor CR, Collins JJ (2000). Construction of a genetic toggle switch in *Escherichia coli*.. Nature.

[20] Hooshangi S, Thiberge S, Weiss R (2005). Ultrasensitivity and noise propagation in a synthetic transcriptional cascade.. Proc Natl Acad Sci USA.

[21] Graumann PL (2006). Different genetic programmes within identical bacteria under identical conditions: the phenomenon of bistability greatly modifies our view on bacterial populations.. Mol Microbiol.

[22] Dubnau D, Losick R (2006). Bistability in bacteria.. Mol Microbiol.

[23] Oppenheim AB, Kobiler O, Stavans J, Court DL, Adhya S (2005). Switches in bacteriophage lambda development.. Annu Rev Genet.

[24] Hasty J, McMillen D, Collins JJ (2002). Engineered gene circuits.. Nature.

[25] Sprinzak D, Elowitz MB (2005). Reconstruction of genetic circuits.. Nature.

[26] Voigt CA (2006). Genetic parts to program bacteria.. Curr Op Biotechnol.

[27] de Lorenzo V, Cases I, Herrero M, Timmis KN (1993). Early and late responses of TOL promoters to pathway inducers: identification of postexponential promoters in *Pseudomonas putida* with *lacZ-tet* bicistronic reporters.. J Bacteriol.

[28] Qazi SN, Harrison SE, Self T, Williams P, Hill PJ (2004). Real-time monitoring of intracellular *Staphylococcus aureus* replication.. J Bacteriol.

[29] Martin L, Che A, Endy D (2009). Gemini, a bifunctional enzymatic and fluorescent reporter of gene expression.. PloS One.

[30] de Lorenzo V (1992). Genetic engineering strategies for environmental applications.. Curr Op Biotechnol.

[31] de Lorenzo V (2008). Systems biology approaches to bioremediation.. Curr Opin Biotechnol.

[32] Jimenez JI, Minambres B, Garcia JL, Diaz E (2002). Genomic analysis of the aromatic catabolic pathways from *Pseudomonas putida* KT2440.. Env Microbiol.

[33] King JM, Digrazia PM, Applegate B, Burlage R, Sanseverino J (1990). Rapid, sensitive bioluminescent reporter technology for naphthalene exposure and biodegradation.. Science.

[34] Galvao TC, de Lorenzo V (2006). Transcriptional regulators a la carte: engineering new effector specificities in bacterial regulatory proteins.. Curr Op Biotechnol.

[35] de Las Heras A, Carreno CA, Martinez-Garcia E, de Lorenzo V (2010). Engineering input/output nodes in prokaryotic regulatory circuits.. FEMS Microbiol Rev.

[36] Kessler B, de Lorenzo V, Timmis KN (1992). A general system to integrate *lacZ* fusions into the chromosomes of gram-negative eubacteria: regulation of the *Pm* promoter of the TOL plasmid studied with all controlling elements in monocopy.. Mol Gen Genet.

[37] Miller WG, Lindow SE (1997). An improved GFP cloning cassette designed for prokaryotic transcriptional fusions.. Gene.

[38] Santos PM, Benndorf D, Sa-Correia I (2004). Insights into *Pseudomonas putida* KT2440 response to phenol-induced stress by quantitative proteomics.. Proteomics.

[39] Visca P, Leoni L, Wilson MJ, Lamont IL (2002). Iron transport and regulation, cell signalling and genomics: lessons from *Escherichia coli* and *Pseudomonas.*. Mol Microbiol.

[40] Harwood CS, Parales RE (1996). The beta-ketoadipate pathway and the biology of self-identity.. Ann Rev Microbiol.

[41] Cowles CE, Nichols NN, Harwood CS (2000). BenR, a XylS homologue, regulates three different pathways of aromatic acid degradation in *Pseudomonas putida.*. J Bacteriol.

[42] Parsek MR, Shinabarger DL, Rothmel RK, Chakrabarty AM (1992). Roles of CatR and cis,cis-muconate in activation of the *catBC* operon, which is involved in benzoate degradation in *Pseudomonas putida.*. J Bacteriol.

[43] Parsek MR, Kivisaar M, Chakrabarty AM (1995). Differential DNA bending introduced by the *Pseudomonas putida* LysR-type regulator, CatR, at the plasmid-borne *pheBA* and chromosomal *catBC* promoters.. Mol Microbiol.

[44] Tropel D, van der Meer JR (2004). Bacterial transcriptional regulators for degradation pathways of aromatic compounds.. Microbiol Mol Biol Revs.

[45] de Lorenzo V, Timmis KN (1994). Analysis and construction of stable phenotypes in Gram-negative bacteria with Tn*5*- and Tn*10*-derived minitransposons.. Meth Enzymol.

[46] Dublanche Y, Michalodimitrakis K, Kummerer N, Foglierini M, Serrano L (2006). Noise in transcription negative feedback loops: simulation and experimental analysis.. Mol Syst Biol.

[47] Choi KH, Gaynor JB, White KG, Lopez C, Bosio CM (2005). A Tn7-based broad-range bacterial cloning and expression system.. Nature Meth.

[48] Herrero M, de Lorenzo V, Timmis KN (1990). Transposon vectors containing non-antibiotic resistance selection markers for cloning and stable chromosomal insertion of foreign genes in gram-negative bacteria.. J Bacteriol.

[49] de Lorenzo V, Herrero M, Metzke M, Timmis KN (1991). An upstream XylR- and IHF-induced nucleoprotein complex regulates the sigma 54-dependent *Pu* promoter of TOL plasmid.. EMBO J.

[50] Perez-Martin J, de Lorenzo V (1995). The sigma 54-dependent promoter *Ps* of the TOL plasmid of *Pseudomonas putida* requires HU for transcriptional activation in vivo by XylR.. J Bacteriol.

[51] Sambrook J, Fritsch EF, Maniatis T (1989). Molecular cloning: A laboratory manual..

[52] de Lorenzo V, Herrero M, Jakubzik U, Timmis KN (1990). Mini-Tn*5* transposon derivatives for insertion mutagenesis, promoter probing, and chromosomal insertion of cloned DNA in Gram-negative eubacteria.. J Bacteriol.

[53] Kristensen CS, Eberl L, Sanchez-Romero JM, Givskov M, Molin S (1995). Site-specific deletions of chromosomally located DNA segments with the multimer resolution system of broad-host-range plasmid RP4.. J Bacteriol.

[54] Miller J (1972). Experiments in molecular genetics..

